# Accuracy of novel anthropometric indices for assessing the risk for progression of prediabetes to diabetes; 13 years of results from Isfahan Cohort Study

**DOI:** 10.20945/2359-4292-2023-0269

**Published:** 2024-10-01

**Authors:** Mohammad Fakhrolmobasheri, Davood Shafie, Behrad Manshaee, Shima Karbasi, Alireza Mazroui, Mahsa Mohammadi Najafabadi, Sadegh Mazaheri-Tehrani, Masoumeh Sadeghi, Hamidreza Roohafza, Maryam Emamimeybodi, Maryam Heidarpour, Najmeh Rabanipour, Nizal Sarrafzadegan

**Affiliations:** 1 Isfahan Cardiovascular Research Center Cardiovascular Research Institute Isfahan University of Medical Sciences Isfahan Iran Isfahan Cardiovascular Research Center, Cardiovascular Research Institute, Isfahan University of Medical Sciences, Isfahan, Iran; 2 Heart Failure Research Center Isfahan Cardiovascular Research Institute Isfahan University of Medical Sciences Isfahan Iran Heart Failure Research Center, Isfahan Cardiovascular Research Institute, Isfahan University of Medical Sciences, Isfahan, Iran; 3 Child Growth and Development Research Center Research Institute for Primordial Prevention of Non-Communicable Disease Isfahan University of Medical Sciences Isfahan Iran Child Growth and Development Research Center, Research Institute for Primordial Prevention of Non-Communicable Disease, Isfahan University of Medical Sciences, Isfahan, Iran; 4 Student Research Committee School of Medicine Isfahan University of Medical Sciences Isfahan Iran Student Research Committee, School of Medicine, Isfahan University of Medical Sciences, Isfahan, Iran; 5 Cardiac Rehabilitation Research Center Isfahan Cardiovascular Research Institute Isfahan University of Medical Sciences Isfahan Iran Cardiac Rehabilitation Research Center, Isfahan Cardiovascular Research Institute, Isfahan University of Medical Sciences, Isfahan, Iran; 6 Cardiac Arrhythmia Center University of California Los Angeles California USA UCLA Cardiac Arrhythmia Center, University of California, Los Angeles, California, USA; 7 Neurocardiology Program of Excellence University of California Los Angeles California USA UCLA Neurocardiology Program of Excellence, University of California, Los Angeles, California, USA; 8 Isfahan Endocrine and Metabolism Research Center Isfahan University of Medical Sciences Isfahan Iran Isfahan Endocrine and Metabolism Research Center, Isfahan University of Medical Sciences, Isfahan, Iran; 9 Department of Biostatistics and Epidemiology, School of Health Isfahan University of Medical Sciences Isfahan Iran Department of Biostatistics and Epidemiology, School of Health, Isfahan University of Medical Sciences, Isfahan, Iran

**Keywords:** Diabetes mellitus, waist circumference, obesity, body mass index, prediabetic state

## Abstract

**Objective:**

We examined the accuracy of novel anthropometric indices in predicting the progression of prediabetes to diabetes.

**Subjects and methods:**

This study was performed on the pre-diabetic sub-population from Isfahan Cohort Study (ICS). Participants were followed up from 2001 to 2013. During every 5-year follow-up survey, patients’ data regarding the incidence and time of incidence of diabetes were recorded. We evaluated the association between the risk of developing diabetes and novel anthropometric indices including: visceral adiposity index (VAI), lipid accumulation products (LAP), deep abdominal adipose tissue (DAAT), abdominal volume index (AVI), A body shape index (ABSI), body roundness index (BRI) and weight-adjusted waist index (WWI). We categorized the indices into two groups according to the median value of each index in the population. We used Cox regression analysis to obtain hazard ratios (HR) using the first group as the reference category and used receiver operating characteristics (ROC) curve analysis for comparing the predictive performance of the indices.

**Results:**

From 215 included subjects, 79 developed diabetes during the 13-year follow-up. AVI, LAP, BRI, and VAI indicated statistically significant HR in crude and adjusted regression models. LAP had the greatest association with the development of diabetes HR = 2.18 (1.36-3.50) in multivariable analysis. ROC curve analysis indicated that LAP has the greatest predictive performance among indices (area under the curve = 0.627).

**Conclusion:**

Regardless of baseline confounding variables, prediabetic patients with a higher LAP index may be at significantly higher risk for developing diabetes.

## INTRODUCTION

The prevalence of type 2 diabetes mellitus (T2DM) is increasing worldwide. The International Diabetes Federation estimates that the prevalence of T2DM may increase to 693 million by 2045 (1). T2DM is a metabolic disorder mainly characterized by hyperglycemia; however, the metabolic derangements in diabetes are far more complicated. Abnormal tissue response to hormonal stimuli, mitochondrial dysfunction, and endothelial dysfunction are associated with the pathophysiology of T2DM (2). Abnormal fat distribution and excessive fat accumulation, generally known as obesity, are strong risk factors for T2DM. Adiposity induces chronic low-grade inflammation, which is the key factor for developing endothelial dysfunction and insulin resistance (IR) (3).

Prediabetes is an intermediate state between diabetes and normal glucose metabolism. The annual progression rate from prediabetes to T2DM is approximately 5% to 10% (4). Obesity is not only a strong risk factor for T2DM but also could accelerate the rate of progression from prediabetes to T2DM. It has been shown that people with a higher body mass index (BMI) have a greater risk for progression to T2DM from prediabetes (5). Moreover, several markers can predict the risk of progression from prediabetes to T2DM. Fasting plasma glucose (FPG), oral glucose tolerance test (OGTT), hemoglobin A1c (HbA1c), fructosamine (FA), glycated albumin (GA), 1.5 anhydroglucitol (1.5 AG), adiponectin, and fetuin-A are all factors reported helping estimate the rate of progression from prediabetes to T2DM (6).

Indicators for glycemic control including FPG, HbA1c, and 2-hours postprandial (2HPP) glucose, are all markers directly associated with serum glucose level. These markers have been reported to be efficient in discriminating high-risk from low-risk patients with prediabetes. Waist circumference (WC), waist to hip ratio (WHR), and BMI are traditional anthropometric indices mainly for discrimination of central obesity (7). WC and WHR are simple and trustworthy measures of abdominal obesity associated with metabolic syndrome (MetS), and some studies indicated the superiority of BMI in predicting MetS (8). In the last decade, new anthropometric indices combining traditional measures with lipid and glucose metabolism biomarkers have been proposed as alternatives to traditional ones. Visceral adiposity index (VAI), lipid accumulation products (LAP) index and deep abdominal adipose tissue (DAAT) are known as non-traditional adiposity indicators expressing obesity and adipose tissue distribution pattern (8-10). LAP and VAI are indices that use both metabolic and anthropometric parameters introduced as sensitive indicators of visceral obesity and practical markers for assessing IR in clinical practice (11). In 2003, the abdominal volume index (AVI) was developed by combining WC and hip circumference (HC) to determine the relationship between abdominal volume and the presence of impaired glucose tolerance (IGT) (12). It has been reported to be one of the strongest anthropometric discriminators for MetS, T2DM, and hypertension (HTN) (13,14). A body shape index (ABSI), which is the WC adjusted for height and weight, was introduced in 2012, appearing to have a strong correlation with premature mortality, T2DM, MetS, and HTN in the general population (15). The body roundness index (BRI) was proposed in 2013 as an indicator of body fat percentage and visceral adipose tissue, which is calculated using simple parameters including WC and height (16). It has been shown to have good discriminatory performance for MetS and HTN in adults from multiple ethnicities (16,17). Recently, weight-adjusted waist index (WWI) was proposed as a new anthropometric index by standardizing WC with body weight. For predicting the incidence of T2DM and HTN, WWI is reported to be the second strong index after BMI (18).

Prediabetes is a condition that has been more investigated lately as a distinct disease (19). That might be due to the fact that, early prevention of T2DM at the stage of prediabetes could effectively reduce the costs of treating patients with T2DM. Studies have indicated that combining lifestyle modification, as the first intervention for preventing T2DM, with pharmacotherapy may effectively help reduce the disease burden and costs for managing T2DM (20). However, there is still no accurate measure to identify individuals with prediabetes who may benefit from more intense lifestyle interventions in order to prevent development of diabetes.

In order to identify the best index for the classification of prediabetic individuals, we investigated the anthropometric indices including BMI, LAP, VAI, DAAT, WWI, WHR, ABSI, BRI, and AVI in predicting the progression of the individual with prediabetes to T2DM.

## SUBJECTS AND METHODS

### STUDY PROTOCOL AND POPULATION

This study was a secondary analysis conducted on a sub-population from Isfahan Cohort Study (ICS). Comprehensive details about the study protocol and participants are reported separately (21,22). ICS is a community-based prospective cohort study with 6,504 participants from January to September 2001 with a follow-up period of 156 months. All participants were individuals aged ≥ 35 selected on a random, multi-stage sampling method from 3 counties in central Iran (Isfahan, Arak, Najafabad). The sample population was recruited to reflect the entire target community’s age, gender, and urban/rural composition. After obtaining the signed informed consent from every individual participating in the study, a comprehensive interview and physical examination were performed. The interview was performed using a predefined questionnaire to collect detailed data surrounding participants’ demographics, socioeconomic status, medical history, family history, physical activity, quality of life, psychological distress, sleeping pattern, dietary habits, and smoking status. Vital signs, cardiopulmonary system, and anthropometric measures were evaluated during the physical examination performed by trained physicians using a predefined checklist. Fasting blood samples were collected to measure fasting plasma glucose, lipid profile, complete blood count, and other laboratory measurements, including C reactive protein (CRP) and erythrocyte sedimentation rate (ESR). Verbal interviews, physical examination, and laboratory measurements were repeated every 5-year in 2007 and 2013 with similar protocols to the beginning of ICS. Outcomes regarding cardiovascular events were evaluated biannually by trained personnel via telephone call interviews using a predefined questionnaire. All participants who were prediabetic at baseline were included in this survey. This study was approved by the ethics committee of Isfahan University of Medical Sciences (approval ID: IR.ARI.MUI.REC.1401.161).

### Outcomes

Prediabetes was defined as fasting blood glucose (FBG) values of 100 to 125 mg/dL or oral glucose tolerance test (OGTT) values of 140 mg/dL or higher and less than 200 mg/dL. At the beginning of the study in 2001, the levels of HbA1c were not measured because the laboratory test for measuring HbA1c was not still widely available. Accordingly, T2DM was defined as any individual with at least two FBG ≥ 126 or 2hPP glucose levels ≥ 200 or on diabetic medications. As mentioned before, during every 5-year follow-up survey, patients’ data regarding the incidence and time of incidence of T2DM were recorded. In addition to the data from the verbal interview, more information pointing toward the time of T2DM onset, including previous blood tests, insurance data, and patient accounts, were used in cases of missing information.

### Measurement

The validated food frequency questionnaire (FFQ) was used to determine the dietary habits (23). The global dietary index (GDI), as a measure of global diet quality, was calculated in which higher scores mean diets higher in total fat, saturated fat, and cholesterol. According to the primary survey of ICS, GDI was found to be an appropriate and feasible index for indicating diet quality among other evaluated indexes (24). The international physical activity questionnaire was used to quantify daily physical activity (25). The Persian version of this questionnaire is formerly validated. All physical examination was performed after 15 minutes of rest in a quiet room, with standard calibrated instruments. Blood pressure (BP) (mmHg) and pulse rate (PR) were measured at 15 minutes intervals. The mean of two measurements was recorded as participants’ BP and PR. The blood pressure was measured by trained physicians using a standardized mercury sphygmomanometer. Height (cm) and weight (kg) were measured using standard calibrated instruments while participants wore thin clothes and no shoes. WC (cm) was measured as the smallest circumference at the costal margin or below. All measurements was performed according to standard protocols (26,27). Similar laboratory measurement methods were used for baseline and follow-ups except for the auto analyzers (Eppendorf, Hamburg, Germany in 2001 vs. Hitachi 902, Japan in 2007), which were tightly qualified and validated by an external standard laboratory center.

### Definition

According to the latest staging for BP by the International Society of Hypertension (ISH), we categorized the BP as follows; normal SBP/DBP (<120 mmHg and <80 mmHg), elevated SBP/DBP (120-129 mmHg and <80 mmHg), stage 1 hypertension (SBP 130-139 mmHg or DBP 80-89 mmHg), stage 2 hypertension (SBP ≥ 140 mmHg or DBP ≥ 90 mmHg) (28). Definition of lipid profile components abnormalities were as total cholesterol (TC) ≥ 200 (mg/dL), low-density lipoprotein-cholesterol (LDL-c) ≥ 160 mg/dL, high-density lipoprotein-cholesterol (HDL-c) < 40 mg/dL in men and < 50 mg/dL in women, and triglycerides (TG) ≥ 150 mg/dL (29). BMI was calculated by dividing weight by the square of height. Other anthropometric indices were calculated according to the formulas listed in the supplementary file, Table S1.

### Statistical analysis

Categorical variables were reported as numbers and percentages in each group. Numerical variables were reported as mean ± standard deviation. We used Pearson’s chi-square and independent sample T-test for categorical and numerical variables to assess between-group differences. Continuous variables (VAI, LAP, BRI, BMI, WC, AVI, DAAT, WHR, WWI, and ABSI) were dichotomized based on median.

We used Cox regression analysis to obtain the crude hazard ratio for each index and forward conditional Cox regression to calculate adjusted hazard ratios. The dependent variable in the cox proportional hazard model was development of diabetes, the independent variables were VAI, LAP, BRI, BMI, WC, AVI, DAAT, WHR, WWI, and ABSI, and the covariates were age, sex, education, GDI, physical activity, FBG, 2hpp, smoking history, lipid profile variables, SBP, and DBP. For Cox proportional analysis, the anthropometric indices were treated as dichotomous variables.

To identify the index with the best predictive performance for development of diabetes from prediabetes, we used the receiver operating characteristics (ROC) curve and calculated the area under the curve for each index. For ROC analysis, the anthropometric indices were treated as continuous variables.

All analysis was performed using SPSS software version 24. The level of significance was considered at P < 0.05.

## RESULTS

**Figure 1:**
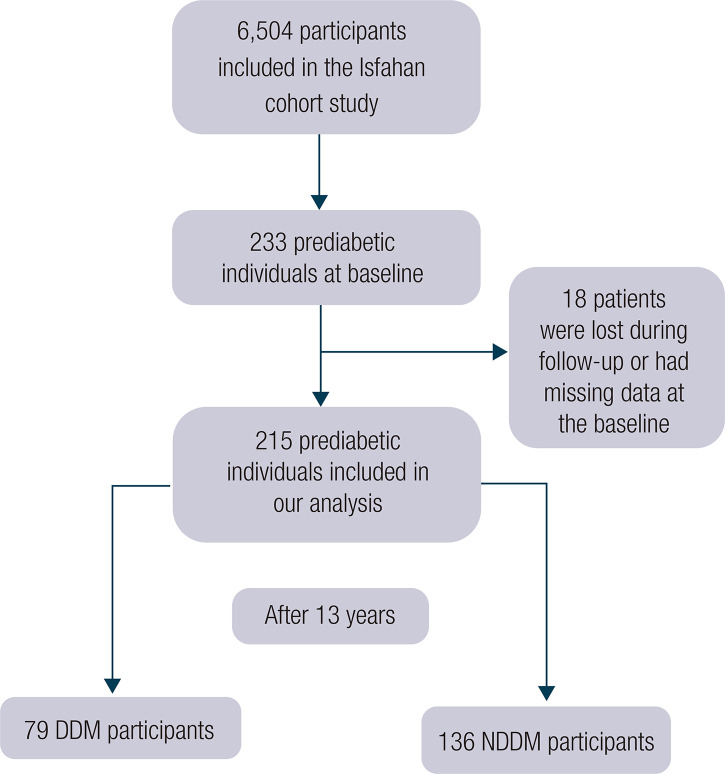
Flow chart of patient inclusion.

From the total included participants in ICS, 233 individuals were prediabetic at the baseline. Eighteen patients were lost to follow-up or had missing data at the baseline; thus, 215 patients were included in our analysis (Figure 1), of which 87 (40.5%) were male. The average age of the population was 50.21 ± 10.42 at the baseline. After 13 years of follow-up, 79 (36.7%) patients developed T2DM, of which 28 (35.4%) were male. There were no significant differences in age, sex, smoking status, education, global dietary index, and total daily physical activity between the participants who developed diabetes mellitus (DDM) and those who did not develop diabetes mellitus (NDDM). There were also no significant differences in the prevalence of high LDL-c, high TG, high TC, and systolic and diastolic HTN between DDM and NDDM groups. However, the DDM group also had a higher weight and waist at the baseline; they had significantly higher FBG, 2hPP, and low HDL-c. Table 1 summarizes the baseline characteristics of the study participants.

**Table 1:** Baseline characteristics of the study population according to the incidence of diabetes

Variables		Total (n=215)	Diabetes		P-value*
			DDM (n=79)	NDDM (n=136)	
Age 1 (year)		50.21±10.42	48.32±9.06	51.30±11.01	0.089
Sex^2^	Male	87 (40.5)	28 (35.4)	59 (43.4)	0.253
	Female	128 (59.5)	51 (64.6)	77 (56.6)	
Weight^1^ (kg)		74.23±11.79	77.06±12.02	72.59±11.37	0.007
Height^1^ (cm)		162±0.97	159.95±10.60	160.31±9.11	0.76
Waist^1^ (cm)		101.21±10.91	103.89±11.04	99.66±10.57	0.003
Education^2^ (year)	0-5	143 (66.5)	49 (62)	94 (69.1)	0.531
	6-12	59 (27.4)	24 (30.4)	35 (25.7)	
	>12	13 (6)	6 (7.6)	7 (5.1)	
Global dietary index^1^		0.97±0.29	1±0.28	0.95±0.29	0.11
Physical activity^1^ (MET-min per week)		846.68±586.67	925.26±610.57	801.03±569.63	0.094
FBG^1^ (mg/dL)		99.57±14.90	106±14.01	95.83±14.15	<0.0001
2hPP glucose^1^ (mg/dL)		152.83±35.83	161.20±35.04	148.12±35.53	0.003
Smoke^2^	Yes	26 (12.1)	10 (12.7)	16 (11.9)	0.862
	No	188 (87.9)	69 (87.3)	119 (88.1)	
Low HDL-c^2^	Yes	110 (51.2)	50 (63.3)	60 (44.1)	0.007
	No	105 (48.8)	29 (36.7)	76 (55.9)	
High LDL-c^2^	Yes	84 (39.1)	35 (44.3)	49 (36)	0.231
	No	131 (60.9)	44 (55.7)	87 (64)	
High TC^2^	Yes	163 (75.8)	62 (78.5)	101 (74.3)	0.486
	No	52 (24.2)	17 (21.5)	35 (25.7)	
High TG^2^	Yes	163 (75.8)	64 (81)	99 (72.8)	0.175
	No	52 (24.2)	15 (19)	37 (27.2)	
Systolic HTN^2^	1	120 (56.1)	46 (59)	74 (54.4)	0.710
	2	9 (4.2)	2 (2.6)	7 (5.1)	
	3	32 (15)	10 (12.8)	22 (16.2)	
	4	53 (24.8)	20 (25.6)	33 (24.3)	
Diastolic HTN^2^	1	152 (71)	57 (73.1)	95 (69.9)	0.802
	2	42 (19.6)	15 (19.2)	27 (19.9)	
	3	20 (9.3)	6 (7.7)	14 (10.3)	

* Independent samples T-test, p value < 0.05 considered significant. ^1^ Continuous variables with normal distribution are summarized as mean ± SD. ^2^ Categorical variables (sex, etc.) are summarized as numbers (%). Abbreviations: DDM: developed diabetes mellitus, NDDM: not developed diabetes mellitus.

The cut-off points discriminating each index into two categories according to the median data are reported in the supplementary file, Table S2.

Table 2 shows the distribution of DDM and NDDM participants among two categories of each anthropometric index. VAI, LAP, BRI, BMI, WC and AVI were significantly associated with the incidence of T2DM. Of 108 individuals in the first group of VAI, 31 persons developed T2DM, whereas, in the second group with 107 subjects, 48 individuals developed T2DM (31/108 vs. 48/107). BRI also indicated a ratio of 29/108 in the below median group vs. 50/107 in the above group. LAP indicated the ratio of 30/108 in the below median group vs. 49/107 in the above median group. These ratios for AVI were 32/108 vs. 47/107 in the below median and above median groups, respectively. BMI indicated a significant difference between groups, with ratios of 32/108 and 47/107 in the below median and above median groups, respectively. Moreover, categorizing the participants according to the WC, demonstrated a significant difference in the progression rate of T2DM (32/110 vs. 47/105 in the below median and above median groups, respectively). Other indices, including ABSI, WWI, WHR, and DAAT, did not indicate any significant difference between the groups regarding the incidence of T2DM.

**Table 2:** Incidence of diabetes in 2 categories in each anthropometric index

Variables	group	Total (n=215)	Diabetes		P-value*
			DDM (n=79)	NDDM (n=136)	
VAI	Below median	108 (50.2)	31 (28.7)	77 (71.3)	**0.0١٤**
	Above median	107 (49.8)	48 (44.8)	59(55.1)	
LAP	Below median	108 (50.2)	30 (27.8)	78 (72.2)	**0.006**
	Above median	107 (49.8)	49 (45.7)	58 (54.3)	
DAAT	Below median	107 (49.8)	39 (36.4)	68 (63.5)	0.93
	Above median	108 (50.2)	40 (37)	68 (63)	
BMI	Below median	108 (50.2)	32 (29.6)	76(70.3)	**0.03**
	Above median	107 (49.8)	47 (43.9)	60 (56.1)	
WHR	Below median	108 (50.2)	38 (48.1)	70 (51.5)	0.63
	Above median	107 (49.8)	41 (51.9)	66 (48.5)	
WWI	Below median	108 (50.2)	37 (46.8)	71 (52.2)	0.45
	Above median	107 (49.8)	42 (53.2)	65 (47.8)	
BRI	Below median	108 (50.2)	29 (26.8)	79 (73.1)	**0.003**
	Above median	107 (49.8)	50 (46.7)	57 (53.2)	
ABSI	Below median	108 (50.2)	44 (40.7)	64 (59.2)	0.22
	Above median	107 (49.8)	35 (32.7)	72 (67.2)	
AVI	Below median	108 (50.2)	32 (29.6)	76 (70.3)	**0.03**
	Above median	107 (49.8)	47 (43.9)	60 (56.1)	
WC	Below median	110 (51.2)	32 (29.1)	78 (70.9)	**0.01**
	Above median	105 (48.8)	47 (44.8)	58 (55.2)	

* Chi-Square Test. p value < 0.05 considered significant. Abbreviations: DDM: developed diabetes mellitus, NDDM: not developed diabetes mellitus.

Results from the Cox proportional hazard ratio (HR) analysis are reported in Table 3. In line with the chi-square test results, AVI, LAP, BRI, WC and VAI indicated statistically significant HR in crude and adjusted regression models. Findings indicated an approximately similar efficacy of BRI and LAP for predicting the incidence of T2DM. Moreover, VAI also indicated a statistically significant predictive value, higher than WC but lower than LAP and BRI. AVI found to have lower predictive power than LAP, BRI, WC and VAI. As shown in the Table 3, the predictive value of the beforementioned indices found to increase after adjustment for cofounding variables. Despite the significant difference in the incidence of T2DM between the below median and above median groups of BMI, crude results from cox regression analysis indicated no significant HR for BMI; however, after adjustment for daily physical activity, low HDL-c, and FBG, the results showed a significant predictive power.

**Table 3:** Crude and adjusted cox regression hazard ratio of prediabetes

Variable	Model	
	Crude	Adjusted
VAI	1.77 (1.13-2.78)	2.10 (1.31-3.36)**
P	**0.013**	**0.002**
LAP	1.85 (1.17-2.91)	2.18 (1.36-3.50)*
P	**0.008**	**0.001**
DAAT	1.028(0.66-1.60)	1.07 (0.67-1.72)*
P	0.90	0.78
BMI	1.541 (0.98-2.41)	1.65 (1.048-2.602)*
P	0.059	**0.031**
WHR	1.096 (0.7-1.7)	0.93 (0.59-1.45)+
P	0.685	0.74
WWI	1.13 (0.72-1.75)	1.19 (0.76-1.86)#
P	0.6	0.46
BRI	1.85 (1.17-2.92)	2 (1.23-3.24)*
P	**0.008**	**0.005**
ABSI	0.76(0.49-1.18)	0.83 (0.53-1.30)+
P	0.22	0.42
AVI	1.62 (1.03-2.54)	1.63 (1.03-2.57)*
P	**0.034**	**0.035**
FBG	1.035 (1.02-1.05)	1.04 (1.02-1.05)++
P	<0.0001	<0.0001
2h PP Glucose	1.01 (1-1.01)	1.01 (1-1.01)++
P	0.021	0.013
Waist Circumference	1.69(1.08-2.65)	1.75 (1.09-2.81)*
P	**0.022**	**0.019**

The first category is a reference for all indexes, but for eGDR the last category is a reference.

* Adjusted for physical activity, low HDL-c, and FBG. ** Adjusted for physical activity, age, and FBG. + Adjusted for weight, low HDL-c, and FBG. # Adjusted for physical activity, low HDL-c, weight, and FBG. ++ Adjusted for low HDL-c, waist, and physical activity. p value < 0.05 considered significant.

**Figure 2:**
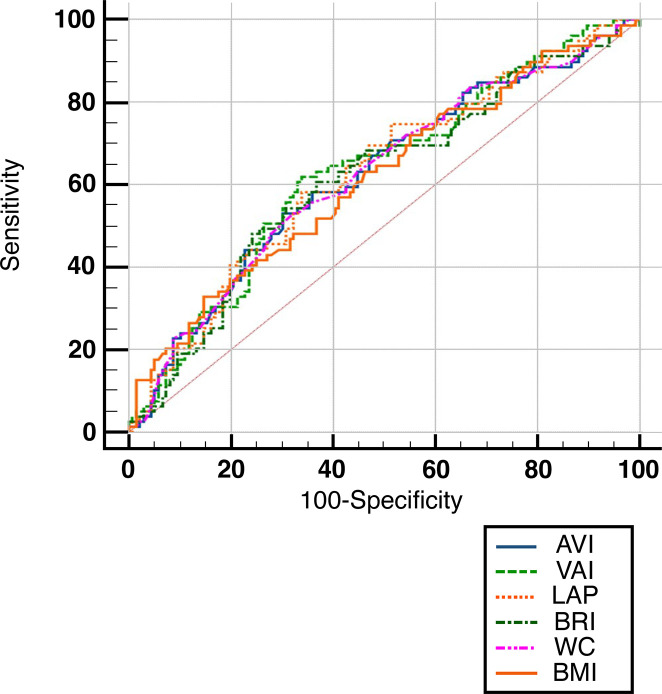
ROC curve for BMI, WC, LAP, VAI, AVI, and BRI according to the development of diabetes.

ROC curve analysis under the non-parametric assumption for indices with statistically significant HRs (Figure 2 and Table 4) indicated that, in line with results from cox regression analysis, LAP has the greatest predictive performance among the other three indices with AUC = 0.627; however, our ROC curve analysis indicates that all the indices have weak predictive power for incidence of T2DM among individuals with prediabetes.

**Table 4:** The area under the curve and 95% confidence interval for indices in the ROC curve

Test Result Variable(s)	Area under the curve	Asymptotic 95% Confidence Interval	
		Lower Bound	Upper Bound
BRI	.615	.536	.694
AVI	.623	.545	.701
BMI	.615	.537	.693
VAI	.616	.539	.693
LAP	.627	.550	.704
WC	.623	.545	.701

## DISCUSSION

To date, few studies have investigated the risk factors associated with the progression of prediabetes to T2DM. In this study on prediabetic individuals from the ICS population, we investigated the association of different anthropometric indices with the rate of progression to T2DM. Our results demonstrated that some of the novel anthropometric indices could be reliable predictors for the progression to T2DM. Besides, we maintain cut-offs for each index in order to better discriminate those at major risk for the development of diabetes. Cox regression analysis unadjusted for baseline confounders showed that VAI, LAP, BRI, and AVI were significantly associated with the risk of prediabetes progression to T2DM. The strongest index in predicting the risk of developing T2DM was LAP. Moreover, in the regression model adjusted for total daily physical activity, low HDL-c, and FBG, LAP was the strongest index associated with the incidence of T2DM. Our results from ROC curve analysis indicated that LAP has the greatest AUC compared to other indices with statistically significant HRs, while AVI, BMI, and BRI had the smallest AUC. LAP has been studied in several studies. LAP is directly associated with insulin resistance in non-diabetic adults (30). Taverna and cols. indicated that LAP is a strong marker of metabolic syndrome in healthy individuals (31). Another study by Malavazos and cols. showed the accuracy of LAP for indicating impaired glucose metabolism (32). In a cross-sectional study by Aline Marcadenti and cols., a positive association between LAP and T2DM in women with HTN was demonstrated (9). Similarly, in the Third National Health and Nutrition Survey Program in the United States, LAP showed a more significant association with T2DM than BMI (33).

Studies have also examined the association between VAI and T2DM. In line with our results, Wang and cols. and Yang and cols. found VAI as a valuable predictor of T2DM. However, they did not indicate the superiority of this index over BMI (34,35). Similar to our results, Ahn and cols. indicated higher ability of LAP in comparison to VAI to discriminate between developing T2DM and not developing T2DM among subjects with prediabetes. These results indicate the close relation between LAP, T2DM, and IR (36).

In contrast to VAI and LAP, our analysis showed that WHR had no significant association with T2DM. Notwithstanding our results, in a cross-sectional study by Li and cols. on a relatively large European sample population, the WHR was significantly associated with an increased risk of T2DM (37). Similarly, in a study by Qureshi and cols., the mean of WHR was significantly higher in the diabetic population. They also demonstrated WHR as a stronger predictor of T2DM compared to BMI (38). These controversial results may be explained by the difference in ethnicities of the studies’ population or different sample sizes. Moreover, different statistical analysis methods may cause controversial results, whereas Qureshi and cols. categorized WHR as below and over 0.88 according to the consensus statement for obesity for Asian Indians and further compared the prevalence of T2DM between study groups. They have also compared the means of WHR between diabetic and non-diabetic patients. Li and cols. using logistic regression models, have reported 1.75 folds higher odds of developing T2DM with one standard deviation increase in WHR. However, in this study, we used cox regression analysis which could yield more reliable results than other methods.

As an index widely accepted for diagnosis of MetS, BMI showed no significant association with the development of T2DM in the crude cox regression model. After adjustment for potential confounders, including physical activity, low HDL-c, and FBG, a significant association with the development of T2DM was observed. In line with our finding, Hellgren and cols. showed that individuals with prediabetes who developed T2DM had higher BMI adjusted for confounding variables (5). Furthermore, in a cohort study by Bennasar and cols. on 27,844 Spanish workers, BMI was identified as a factor associated with the progression of prediabetes to T2DM (39).

WC as another traditional index that extensively used in the diagnosis of metabolic disorders, showed a significant predictive value for T2DM development. In line with our results, Nakanga and cols. demonstrated that WC has a significant predictive value for T2DM progression in people with prediabetes in sub-Saharan Africa (40). Moreover, WC showed to be a significant predictor for T2DM incidence among Iranian population, even slightly better than BMI (41,42).

WWI is one of the recently developed indices based on WC adjusted for weight. This index is reported to predict cardiovascular mortality better than BMI and WC (18). While higher WWI appears to be strongly associated with an increased risk for HTN, it has been shown that WWI has limited ability to discriminate MetS in non-overweight/obese adults (43,44). However, the discriminating value of WWI for developing diabetes from prediabetes, has been not studied before, and our study found no statistically significant correlation between high WWI and the rate of T2DM development in both crude and adjusted models.

Thomas and cols. were the first to propose BRI based on height and WC to estimate body fat percentage (45). Studies have indicated that BRI is strongly associated with HTN. BRI showed a significantly stronger correlation with HTN rather than ABSI (17,44). Additionally, BRI is strongly associated with arterial stiffness compared to classic anthropometric indices and seems to be the best predictor for Coronary Heart Diseases (CHD) in females. However, the same study suggested ABSI as the best predictor for CHD in males (46,47). In a recent meta-analysis, it was concluded that BRI has the superiority in predicting MetS compering to BMI, WHR, and ABSI (17). Studies suggested a high power for BRI in predicting T2DM among Japanese population (48,49), and hypertensive Chinese patients (50). However, none of them evaluated the prediabetic population. In the current study, we found that BRI is one of the indices which could independently predict prediabetes progression to T2DM, while ABSI did not indicate any significant association.

AVI is another index significantly associated with an increased risk of T2DM progression in this study. AVI was first proposed by Guerrero-Romero and cols. for determining the risk of T2DM, where it is derived from HC and WC, and it showed to be strongly related to IGT and T2DM (12). Recently, it has been concluded that AVI is the optimal index to identify MetS and a good predictor of HTN (13,44).

Taken together, our results indicated that novel anthropometric indices could effectively predict the risk of developing T2DM in individuals with prediabetes. Among indices evaluated in our study VAI, LAP, AVI, BMI, and BRI were significantly associated with the development of T2DM. Among them, LAP had the strongest ability to predict the incidence of T2DM. In clinical practice maintaining cut-off points of mentioned indices may help to find people at higher risk of diabetes as an add-on way to the glycemic profile.

### Limitations and strengths

In this study, we faced many limitations. The sample population of ICS was selected from healthy individuals without any history of cardiovascular disorders, and the recruitment of participants was not based on prediabetes. Our results were according to a cohort study of a relatively small population; thus, we suggest further research in this field regarding the fact that there is limited data on risk stratification of the prediabetic population. Moreover, at the beginning of the study in 2001, laboratory test for measuring HbA1c was not still widely available, and the levels of HbA1c were not measured in the study participant. In this regard, we had to limit the definition of prediabetes and T2DM to the markers of FBG and 2HPP. Using survival analysis with data according to the time of onset of T2DM besides long-term follow-up of study participants and relatively low percentage of the population with missing data were the points of strength in this study.

In conclusion, simple methods for risk stratification of prediabetic patients could effectively help identify individuals who may benefit more from more intense lifestyle interventions to reverse the pathogenesis of T2DM. Novel anthropometric indices, independent of glycemic control biomarkers, could help discriminate high- from low-risk prediabetic individuals. Thus, to better distinguish high-risk cases, we can utilize these indices as an add to glycemic markers. LAP has the most vital ability to predict the development of T2DM from prediabetes. VAI, BRI, and AVI are indices with a lower ability to predict progression to T2DM.
