# Supplementary material for: Accuracy of novel anthropometric indices for assessing the risk for progression of prediabetes to diabetes; 13 years of results from Isfahan Cohort Study
Source: Arch Endocrinol Metab. 2024 Oct 1;68:e230269. doi: 10.20945/2359-4292-2023-0269 (PMC11460962; doi:10.20945/2359-4292-2023-0269)
Supplement: Supplementary file 1 [file 2359-4292-aem-68-e230269-suppl01.pdf]

SUPPLEMENTARY

Table S1. The formula used for calculating anthropometric indices

index		Formula
LAP[29]	Male	$(\mathrm{WC} - 65) \times \mathrm{TG}$
	Female	$(\mathrm{WC} - 58) \times \mathrm{TG}$
VAI[11]	Male	$\left(\frac{WC}{39.68 + BMI \times 1.88}\right) \times \left(\frac{total\ TG}{1.03}\right) \times \frac{1.31}{HDL}$
	Female	$\left(\frac{WC}{36.58 + BMI \times 1.89}\right) \times \left(\frac{total\ TG}{0.81}\right) \times \frac{1.52}{HDL}$
DAAT[11]	Male	$(1.09 \times \mathrm{weight}) + (6.04 \times \mathrm{WC}) - (2.29 \times BMI) - 382.9$
	Female	$(-0.86 \times \mathrm{weight}) + (5.19 \times \mathrm{WC}) - 278$
BRI[30]		$364.2 - 365.5 \times \sqrt{1 - \left(\frac{\frac{WC}{2\pi}}{height \times 0.5}\right)^2}$
WWI[20]		$\frac{WC}{\sqrt{weight}}$
AVI[14]		$\frac{WC^2 \times 2 + (WC - hip)^2 \times 0.7}{1000}$
ABSI[31]		$\frac{WC}{\sqrt[3]{BMI^2} \times \sqrt{height}}$

WC, waist circumference; TG, triglyceride; BMI, body mass index; HDL-c, high-density lipoprotein cholesterol; LAP, lipid accumulation products; VAI, visceral adiposity index; DAAT, deep abdominal adipose tissue; BRI, body roundness index; WWI, weight-adjusted waist index; AVI, abdominal volume index; ABSI, a body shape index. Weight, length, and waist measurements are in order in kg, m, and cm.

Table S2. Median values used for categorizing each index

	BMI	WHR	WWI	BRI	ABSI	AVI	LAP	DAAT	VAI
Median	28.5483	.9554	11.6276	6.1117	.8672	20.4132	90.2071	156.4505	3.3679

Copyright© AE&M all rights reserved.
